# Three-Dimensional Anatomic Evaluation of the Anterior Cruciate Ligament for Planning Reconstruction

**DOI:** 10.1155/2012/569704

**Published:** 2011-10-05

**Authors:** Yuichi Hoshino, Donghwi Kim, Freddie H. Fu

**Affiliations:** Department of Orthopaedic Surgery, University of Pittsburgh, Pittsburgh, PA 15213, USA

## Abstract

Anatomic study related to the anterior cruciate ligament (ACL) reconstruction surgery has been developed in accordance with the progress of imaging technology. Advances in imaging techniques, especially the move from two-dimensional (2D) to three-dimensional (3D) image analysis, substantially contribute to anatomic understanding and its application to advanced ACL reconstruction surgery. This paper introduces previous research about image analysis of the ACL anatomy and its application to ACL reconstruction surgery. Crucial bony landmarks for the accurate placement of the ACL graft can be identified by 3D imaging technique. Additionally, 3D-CT analysis of the ACL insertion site anatomy provides better and more consistent evaluation than conventional “clock-face” reference and roentgenologic quadrant method. Since the human anatomy has a complex three-dimensional structure, further anatomic research using three-dimensional imaging analysis and its clinical application by navigation system or other technologies is warranted for the improvement of the ACL reconstruction.

## 1. Introduction

Recent progress of the anterior cruciate ligament (ACL) reconstruction procedure and related research largely stems from the increased attention to the restoration of the original anatomy. An accurate evaluation of the native anatomy is critical for achieving anatomic ACL reconstruction. Clinical outcome could be imperfect when the graft placement is not located at an anatomic position [1, 2]. Also, conventional transtibial ACL reconstruction, which often locates the graft away from anatomic location [3], leads to abnormal biomechanical behavior and in vivo knee kinematics [4–6], which could influence long-term knee joint health [7, 8]. On the other hand, the anatomical ACL reconstruction procedure, either single-bundle or double-bundle technique, could provide better knee kinematics than nonanatomic reconstruction [9–11] and promising clinical results [12–20]. Appropriate anatomic evaluation of the native ACL for each individual patient can provide critical information for planning ACL reconstruction in an anatomic fashion, while postoperative evaluation of the reconstructed ACL graft location could predict the prognosis after the surgery and give valuable feedback to surgeons. In theory, a conventional two-dimensional assessment cannot fully recognize the three-dimensional structure of the original anatomy. Therefore, three-dimensional imaging analysis of the knee has been progressively developed over the last few years [21, 22]. 

The purpose of this paper is to introduce the progression of imaging technology into three-dimensional analysis on various fields of research related to the ACL and to suggest the future direction of ACL-related anatomic studies.

## 2. Progression of the Imaging Technology for the ACL Anatomy

### 2.1. Image Analysis for the ACL Insertion Site

Anatomic research investigating the native ACL location has been developed over the last several decades. In 1975, Girgis et al. [23] investigated the cruciate ligaments in cadaveric knees focusing on their function and first recognized the different functional bundles of the ACL, the anteromedial (AM) and posterolateral (PL) bundles [23]. The relative positions between the two bundles were identified, while the exact locations were not determined due to lack of baseline anatomic landmarks and scales. In the 1980s, relative distances from the anatomic landmarks, such as the anterior edge of the intercondylar notch, lateral condyle surface, and cartilage margin, on a hypothetical sagittal plane which was usually set on the lateral wall of the intercondylar notch were often used for describing the anatomical location. One of the examples of the two-dimensional anatomic description on the lateral wall of the intercondylar notch is the isometric mapping introduced by Sidles et al., which showed the relationship between the anatomical ACL graft location on a hypothetical plane and the length change of the graft [24]. They revealed that the original ACL insertion site is different from the isometric point where the graft does not change length during flexion-extension movement, whereas some cadaveric experiments at that time demonstrated that the anatomical location of the original ACL could produce the isometric length change pattern of the ACL graft [25, 26]. This disparity of the results could be caused by lack of common anatomic definition. In the 1990s, two-dimensional anatomical scales, such as the “clock-face” reference and quadrant method, were developed as a fixed and universal scaling method to describe the anatomical location of the ACL insertion site and graft placement. The “clock-face” reference has been utilized for referring the coronal position of the ACL insertion site and graft placement [27–29]. This reference system is able to be adopted for arthroscopic images [30]. However, a recent study shows that the interobserver variability is still high using the clock-face reference [31]. Interestingly, Siebold et al. demonstrated that the AM and PL were aligned at the same level on the “clock-face” reference, at 1:30 o'clock, when the knee flexed at 102 degrees [32]. It reversely implied that the anatomical location indicated by the clock-face reference can be easily modified by changing knee flexion angle. This critical flaw of the “clock-face” reference has been frequently pointed out [33, 34]. In the meantime, the quadrant method, originally described by Bernard et al. [35], is the most commonly used reference for the location of the ACL on the lateral X-ray of the distal femur [36–38]. However, this system depends crucially on an unstable baseline, Blumensaat's line, which is a projected line of the intercondylar roof on the lateral X-ray of the distal femur. Farrow et al. [39] demonstrated by cadaver experiment that the posterior edge of Blumensaat's line cannot be consistently recognized [39]. Also, Berg et al. [40] reported the large variability in the angle measurement of Blumensaat's line against the femoral shaft [40]. In addition, the anatomical location indicated by this system cannot be reproduced under arthroscopy. Also, the true lateral view of the distal femur is also extremely difficult to achieve by intraoperative fluoroscopy. Therefore, clinical value of this technique is assumed to be limited. 

As anatomic double bundle ACL reconstruction was introduced in the 2000s, meticulous investigations of bony landmarks were conducted to identify useful landmarks which are available under arthroscopy and can be used for precise graft placements. Three-dimensional (3D) CT images contributed to this decade's progress of anatomic research. Purnell et al. [41] demonstrated that the lateral intercondylar ridge, formally known as “resident's ridge” [42], was clearly shown by the 3D CT image and can be defined as the anterior edge of the ACL original insertion site [41]. Furthermore, Ferretti et al. [43] observed the bifurcate ridge on the lower third of the lateral wall which separates the AM and PL bundles [43], which can also be recognized by 3D CT image [44]. Although these tiny bony ridges are not always visible under arthroscopy [45], these osseous landmarks provides useful information for identifying the original ACL insertion site and performing the ACL reconstruction in an anatomic fashion [44]. Further advantage of the three-dimensional CT is the ability to arrange the rotation of the 3D image in a standardized orientation, providing consistent mapping of the ACL tunnel location [21]. This technique can be used for accurate and repeatable analysis of the ACL graft tunnel locations after reconstruction [3]. 

The progress of CT imaging technique from two- to three-dimensional has contributed significantly to the anatomic research of the ACL. Similar progress is still warranted for MRI because of its capacity to evaluate soft-tissue structures.

### 2.2. Imaging Analysis for Intercondylar Notch Geometry

Femoral intercondylar notch width has been often discussed as a risk factor for the ACL injury, but it remains unknown if the narrow notch truly leads to ACL injury. Several studies evaluating intercondylar notch width by X-ray or CT reported narrow notch as a risk factor for the ACL injury [46–52]. Houseworth et al. [47] measured notch width using notch view, which is an AP view of the knee joint with 45 degrees of knee flexion, and demonstrated a correlation between femoral intercondylar notch stenosis and anterior cruciate ligament injuries [50]. However, other research cast doubt on the impact of the intercondylar notch stenosis [53, 54]. Schickendantz and Weiker [53] prospectively measured the notch width in professional basketball players, and their follow-up survey did not find significant difference of the notch width between ACL injured and noninjured players [53]. Those ambivalent results might be caused by the two-dimensional measurement of the intercondylar notch width. Van Eck et al. [55] compared two-dimensional and three-dimensional measurement of intercondylar notch geometry and demonstrated that there were only moderate correlations between those two measurement [55]. Since X-ray measurement is largely influenced by rotation and angulations [52], three-dimensional measurement is preferable to assess the intercondylar notch geometry. 

### 2.3. Application to Navigation System for the ACL Reconstruction

The development of 3D imaging techniques and increased attention to the anatomic procedure are accompanied with the advanced technology of computers and robots, leading to strong motivation for computer-assisted surgery which could provide real-time imaging feedback with the individualized anatomic information to the surgeons during ACL reconstruction.

In the early 1980s, computer-assisted ACL reconstruction was first attempted by the technique using stereotaxic frame of CT scan for brain surgery [56]. However, it was not commercialized due to excessive cost, invasiveness, and especially prolonged operation time due to computer processing. An imageless navigation system was introduced in the mid-1990s [57], while anatomic placement drew increased attention because of higher revision rate as much as 10% to 40% due to inaccurate tunnel placement [58]. However, navigation-assisted ACL reconstruction was not accepted even though it had inherent accuracy mainly due to the invasiveness by fixing the tracker or exposing radiation and poor cost effectiveness [59]. Meanwhile, experimental use of the navigation system has flourished as an experimental tool. According to Zaffagnini et al., published research using navigation systems could be divided in two groups: anatomical studies (ligament insertion, tunnel position, graft isometry, and impingement) and kinematic studies (Lachman test, anterior drawer test, internal rotation and external rotation, and pivot shift test) [59]. Anatomic studies focused on the evaluation of graft length change and graft impingement against the intercondylar notch roof after ACL reconstruction [57] and the validation of the system accuracy [60], whereas knee kinematics evaluation by navigation system was largely performed to compare various operation techniques [61–64]. 

Navigation systems can be divided into image-guided [65] and imageless systems [56]. In an image-guided system, preoperative images from fluoroscopy, CT, or MRI are inputted and utilized to provide real-time feedback of anatomic information under arthroscopy. In imageless system, which is more common, positional information of anatomical landmarks and joint kinematics is intraoperatively registered for providing anatomic feedback. Nakagawa et al. [65] converted intraoperative 2D fluoroscopic picture into 3D image for preventing blow-out fractures while drilling the femoral tunnel [65]. However, their 2D-3D matching technique did not identify either lateral intercondylar ridge or bifurcate ridge [65].

Navigation systems can provide real-time quantitative feedback during arthroscopic procedure and minimize technical error with enhanced reproducibility and reliability [56, 66]. However, these systems must be improved before wider acceptance of the navigation system, such as invasiveness, cost effectiveness, and complexity of the navigation processing. Moreover, clear and reproducible definition of ideal tunnel position based on concrete anatomic baseline should be established to restore normal joint kinematics after ACL reconstruction.

## 3. Conclusion

Research on the anatomy of to the ACL has progressed along with the advancement of imaging and navigation technology. Useful bony landmarks for placing the ACL graft at the ideal position can be identified by 3D imaging technique. Also, 3D-CT analysis for the location of the native and reconstructed ACL provides better and more consistent evaluation than conventional “clock-face” reference and roentgenologic quadrant method. Three-dimensional image analysis of the ACL anatomy and its application to the navigation system is becoming more prevalent and reliable for advancing the anatomic studies related to the native ACL and the ACL reconstruction procedure.
